# Supplementary material for: In vivo metabolic tagging and targeting of circulating red blood cells
Source: Nat Commun. 2026 Mar 21;17:4298. doi: 10.1038/s41467-026-71013-x (PMC13172334; doi:10.1038/s41467-026-71013-x)
Supplement: Supplementary file 1 — Supplementary Information [file 41467_2026_71013_MOESM1_ESM.pdf]

Supplementary Figures for

In Vivo Metabolic Tagging and Targeting of Circulating Red Blood Cells

Yusheng Liu[1], Yizun Wang[2], Kyungwon Ko[1], Yuan Liu[1], Haiyi Huang[3], Yueji Wang[1,4], Jiadiao Zhou[1], Dhyanesh Baskaran[1], Joonsu Han[1], Rimsha Bhatta[1], Daniel Nguyen[1,2], Cecilia Leal[1,2,5,6,7], Matthew R. Berry[8], Fan Lam[2,5,6,9,10,11], Hua Wang[1,2,5,6,7,10,11]*

[1]Department of Materials Science and Engineering, University of Illinois at Urbana-Champaign, Urbana, IL 61801, USA. [2]Department of Bioengineering, University of Illinois at Urbana-Champaign, Urbana, IL 61801, USA. [3]Department of Chemistry, University of Illinois at Urbana-Champaign, Urbana, IL 61801, USA. [4]Department of Mechanical Science and Engineering, University of Illinois at Urbana-Champaign, Urbana, IL 61801, USA.[5]Carle College of Medicine, University of Illinois at Urbana-Champaign, Urbana, IL 61801, USA. [6]Beckman Institute for Advanced Science and Technology, University of Illinois at Urbana-Champaign, Urbana, IL 61801, USA. [7]Materials Research Laboratory, University of Illinois at Urbana-Champaign, Urbana, IL 61801, USA. [8]Department of Veterinary Clinical Medicine, University of Illinois at Urbana-Champaign, Urbana, IL 61802, USA. [9]Department of Electrical and Computer Engineering, University of Illinois at Urbana-Champaign, Urbana, IL 61801, USA. [10]Cancer Center at Illinois (CCIL), Urbana, IL 61801, USA. [11]Institute for Genomic Biology, University of Illinois at Urbana-Champaign, Urbana, IL 61801, USA.

*Correspondence should be addressed to huawang3@illinois.edu

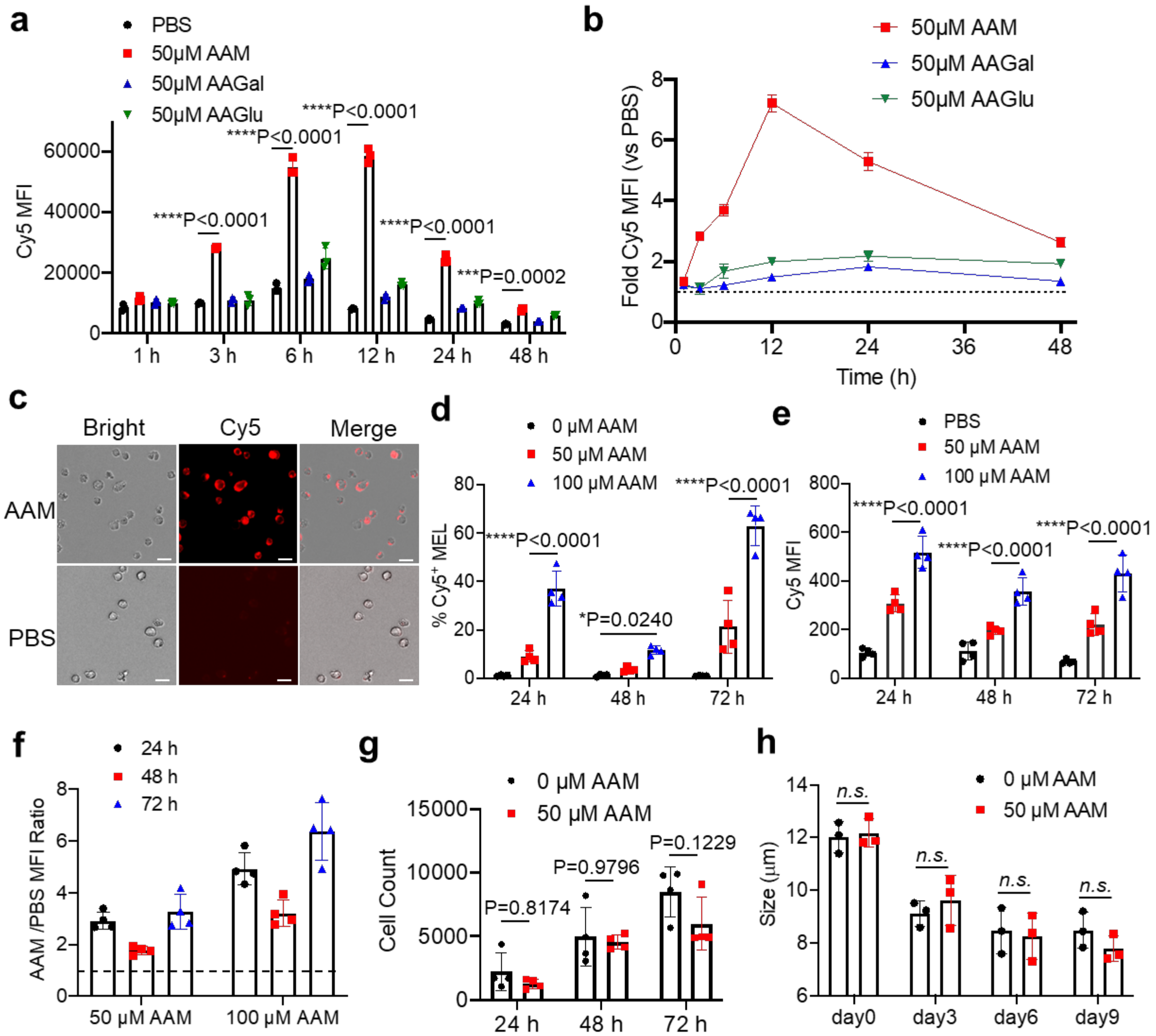

Supplementary Figure 1. Metabolic glycan labeling of MEL cells. (a) Mean Cy5 fluorescence intensity of MEL cells after incubation with azido-sugars or PBS for varied time (1, 3, 6, 12, 24, or 48 h) and staining with DBCO-Cy5 for 1 h. A concentration of 50 μM was used for all azido-sugars (AAM, AAGal, or AAGlu, n=3 samples). (b) Cy5 fluorescence intensity ratio of MEL cells (azido-sugar/PBS) after incubation with azido-sugars for 1, 3, 6, 12, 24, and 48 h, respectively. (c) Fluorescence images of MEL cells treated with AAM for 24 h and then incubated with DBCO-Cy5 for 1 h. Scale bar: 10 μm. (d-g) MEL cells were treated with different concentrations of AAM for 24, 48 and 72 h, respectively, with fresh AAM added at 48 h (n=4 samples per group). Shown are (d) % Cy5^{+} MEL cells, (e) mean Cy5 fluorescence of MEL cells, (f) Cy5 fluorescence ratio of MEL cells (AAM/PBS), and (g) counts of MEL cells after azido-sugar treatment. (h) Size of cells during the differentiation process of MEL cells in the presence or absence of AAM (n=3 samples). All the numerical data are presented as mean ± SD (one-way ANOVA with post hoc Fisher's LSD test was used; 0.01 < *P ≤ 0.05; **P ≤ 0.01; ***P ≤ 0.001; ****P ≤ 0.0001). Experiment in (c) was performed at least three times independently with similar results. Source data for this figure is available in the Source Data file.

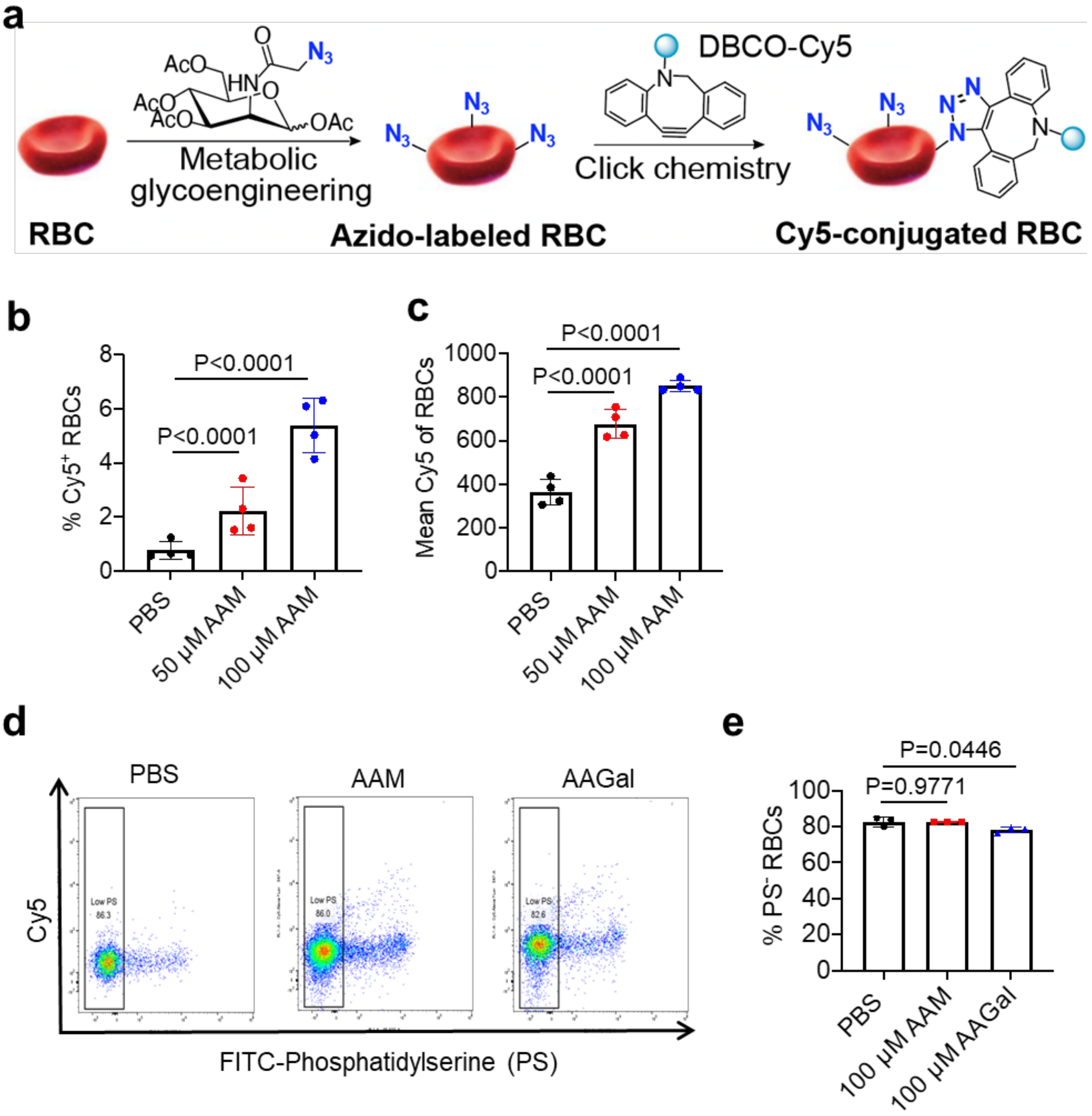

Supplementary Figure 2. Metabolic glycan labeling of mouse RBCs in vitro. (a) Schematic illustration of metabolic labeling of mouse RBCs with azido groups and subsequent conjugation of DBCO-molecules via click chemistry. (b) % $Cy5^+$ RBCs and (c) mean Cy5 fluorescence intensity of RBCs after 24-h incubation with AAM or PBS (n=4 samples per group) and 1-h staining with DBCO-Cy5. (d) Representative phosphatidylserine (PS) histogram of mouse RBCs after 24-h incubation with AAM or AAGal or PBS. (e) % PS^+ RBCs after 24-h incubation with AAM or AAGal or PBS (n=3 samples per group). All the numerical data are presented as mean ± SD (one-way ANOVA with post hoc Fisher's LSD test was used; $0.01 < {*}P \leq 0.05$; $^{**}P \leq 0.01$; $^{***}P \leq 0.001$; $^{****}P \leq 0.0001$). Source data for this figure is available in the Source Data file.

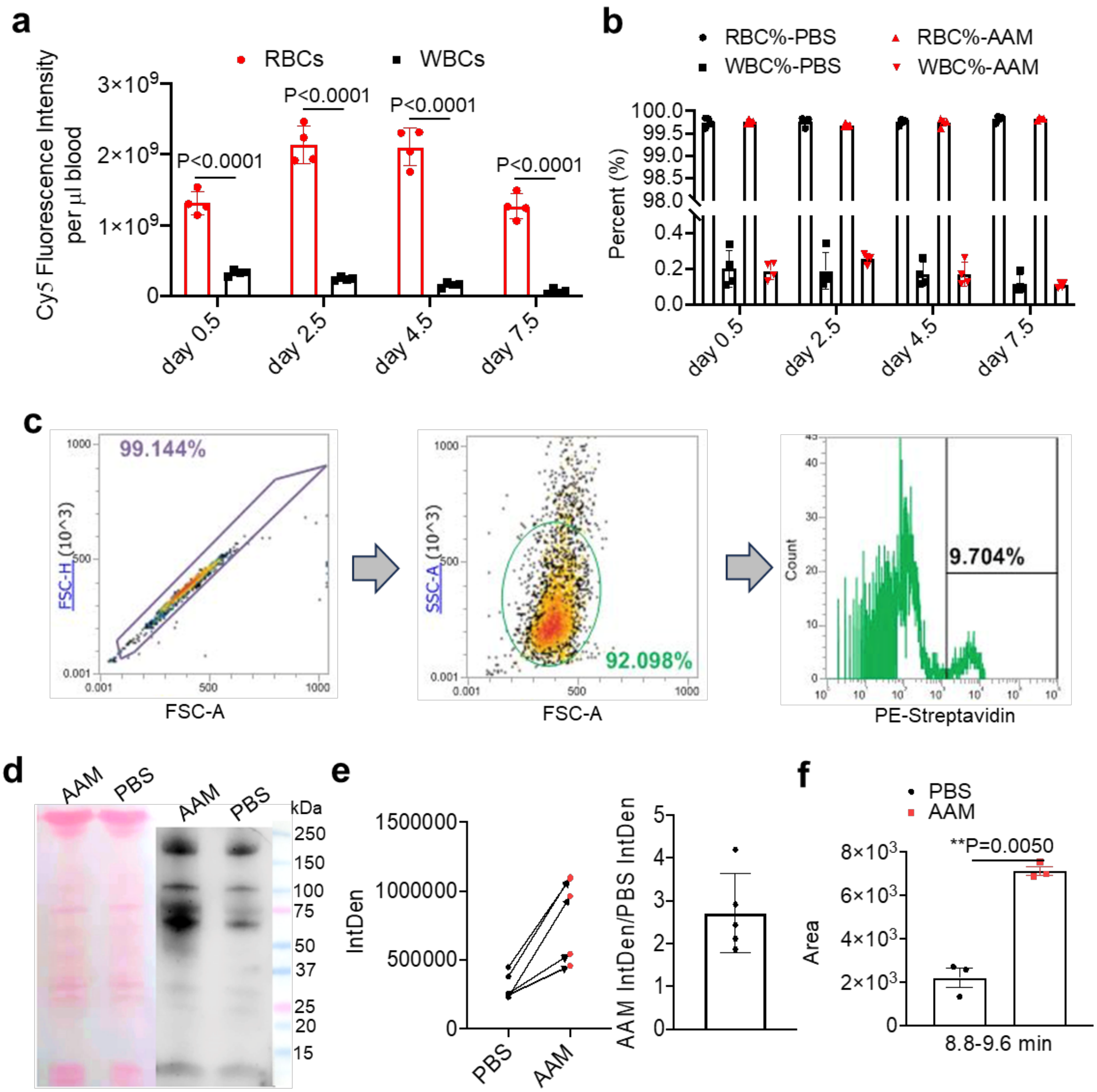

Supplementary Figure 3. AAM can metabolically label glycoproteins and glycolipids of RBCs. C57BL/6 mice were i.v. injected with AAM or PBS twice daily for three days. (a) Total Cy5 fluorescence intensity of RBCs and WBCs that were harvested at different times and stained with DBCO-Cy5, as calculated by # of RBCs (or WBCs) per µL × mean Cy5 fluorescence intensity of RBCs (or WBCs) (n= 4 mice per group). (b) Percentages of RBCs and WBCs among blood cells at different times post injections of AAM or PBS (n=4 mice per group). (c) Representative flow plots for analyzing azido-labeled RBCs. (d) Western blot analysis of RBCs isolated from mice at 14 days post AAM or PBS injections. Proteins extracted from RBCs were incubated with DBCO-biotin, run on a gel, and detected with streptavidin-HRP. (e) Quantification of 5 protein band signal change in (d). (f) Quantification of Cy5-conjugated lipids from HPLC profiles (n= 3 samples, shown in Main Figure 2d). All the numerical data are presented as mean ± SD (two-tailed Welch's t-test was used; 0.01 < *P ≤ 0.05; **P ≤ 0.01; ***P ≤ 0.001; ****P ≤ 0.0001). Experiment in (d) was performed at least three times independently with similar results. Source data for this figure is available in the Source Data file.

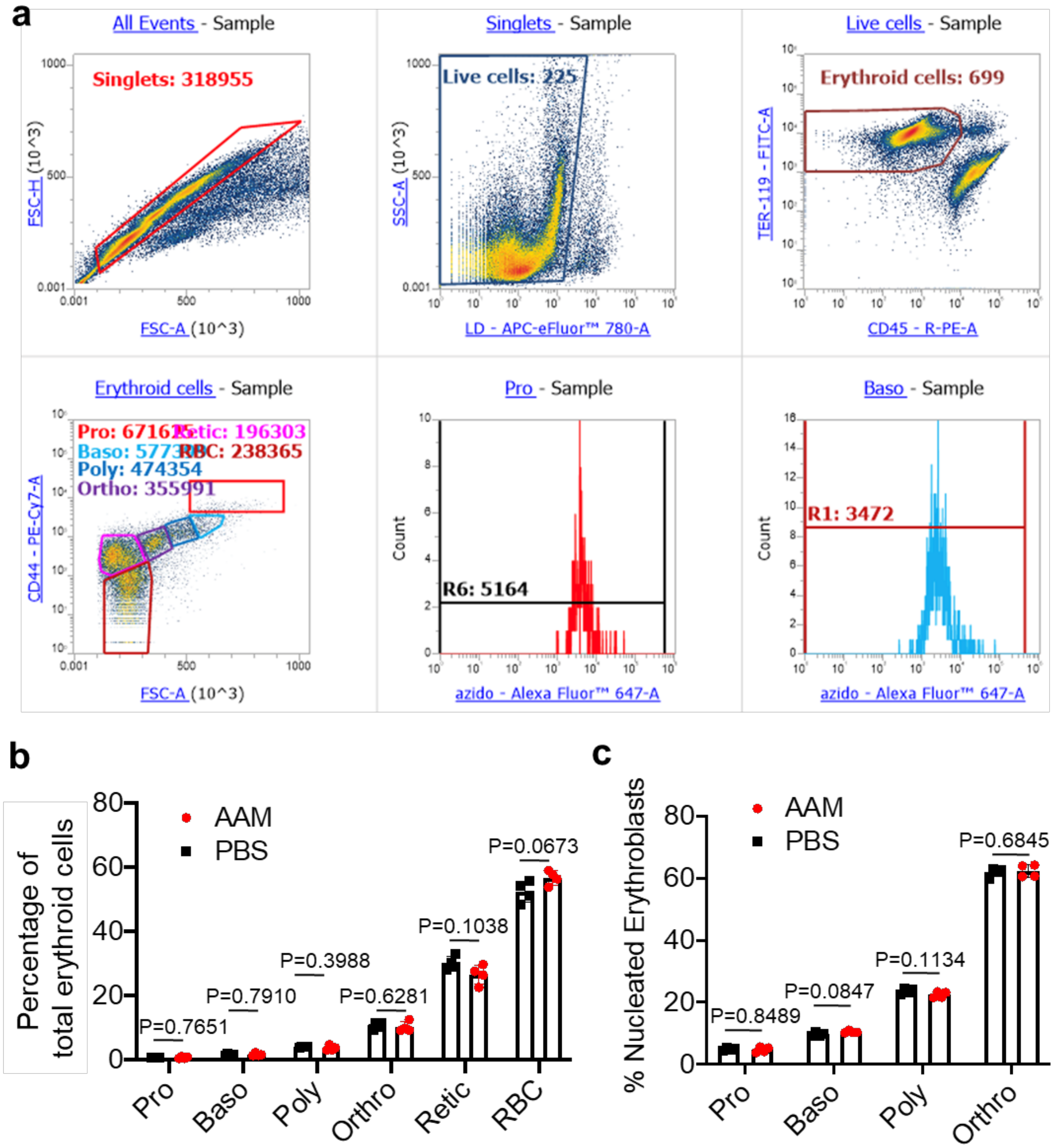

Supplementary Figure 4. Intravenously injected AAM successfully labels RBC precursor cells in the bone marrow. C57BL/6 mice were i.v. injected with AAM or PBS twice daily for three days. Bone marrow was harvested at 48 h post injections of AAM or PBS. (a) Representative flow plots for analyzing different erythroid lineage cells in the bone marrow. (b) Percentages of different RBC precursor cells among total erythroid cells at 2 days post injections of AAM or PBS (n=4 mice per group). (c) Percentages of nucleated erythroid lineage cells in the bone marrow at 2 days post injections of AAM or PBS (n=4 mice per group). All the numerical data are presented as mean ± SD (one-way ANOVA with post hoc Fisher's LSD test was used; $0.01 < {*}P \leq 0.05$; $^{**}P \leq 0.01$; $^{***}P \leq 0.001$; $^{****}P \leq 0.0001$). Source data for this figure is available in the Source Data file.

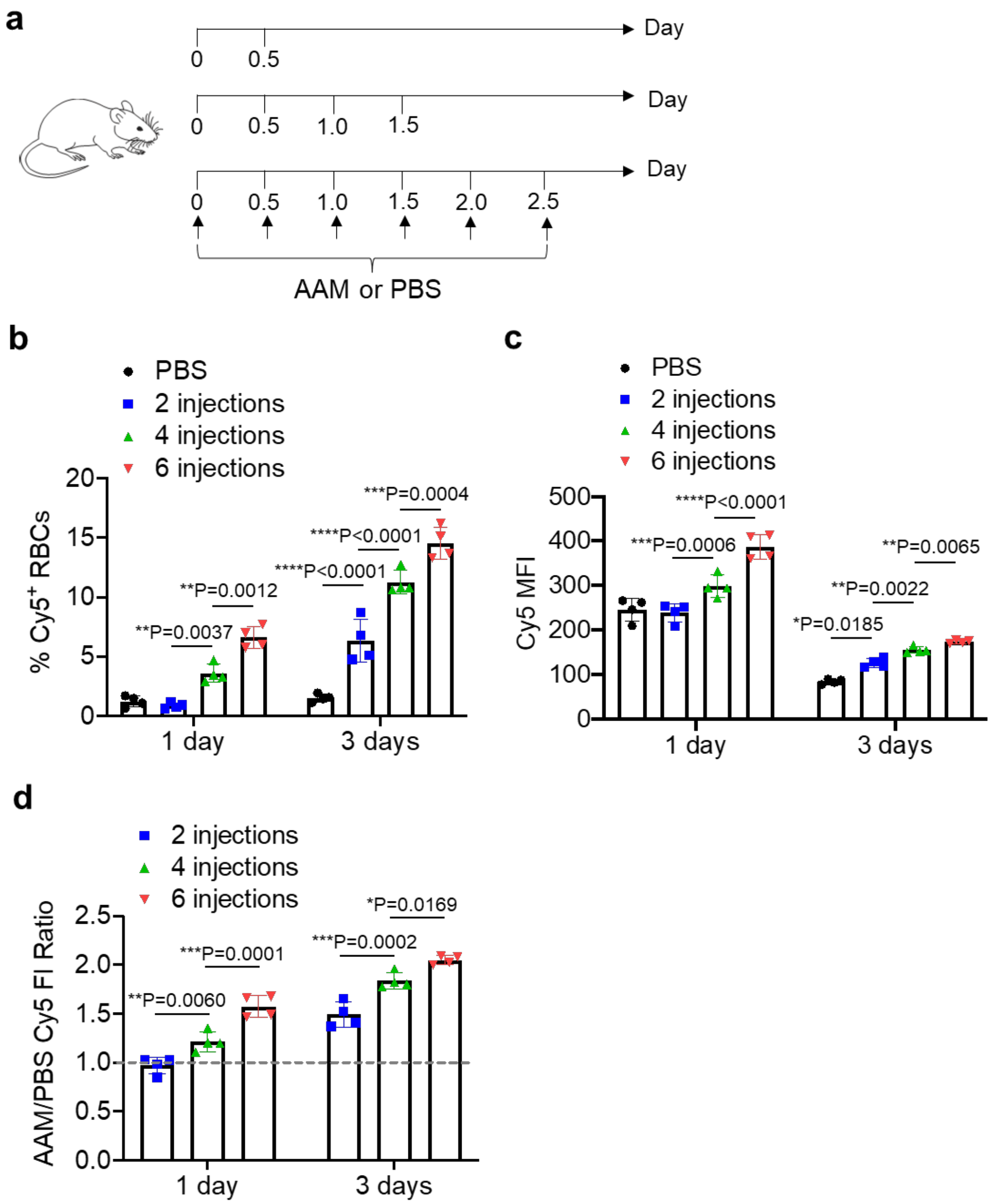

Supplementary Figure 5. Intraperitoneally injected AAM metabolically labels RBCs with azido groups in vivo in a dose frequency dependent manner. (a) Timeframe for the in vivo RBC labeling study. AAM (200 mg/kg) or PBS was intraperitoneally injected for 0, 2, 4, and 6 times, respectively with an interval of 12 h. RBCs were isolated at different times and incubated with DBCO-Cy5 for the detection of cell-surface azido groups. (b) Percentages of $Cy5^{+}$ RBCs at 1 or 3 days post the last injection of AAM or PBS. (c) Mean Cy5 fluorescence intensity of RBCs at 1 or 3 days post the last injection of AAM or PBS. (d) Cy5 fluorescence intensity ratio of RBCs (AAM/PBS) in (c). For (b-d), n=4 mice per group. All the numerical data are presented as mean ± SD (one-way ANOVA with post hoc Fisher's LSD test was used; $0.01 < *P \leq 0.05$; $**P \leq 0.01$; $***P \leq 0.001$; $****P \leq 0.0001$). Source data for this figure is available in the Source Data file.

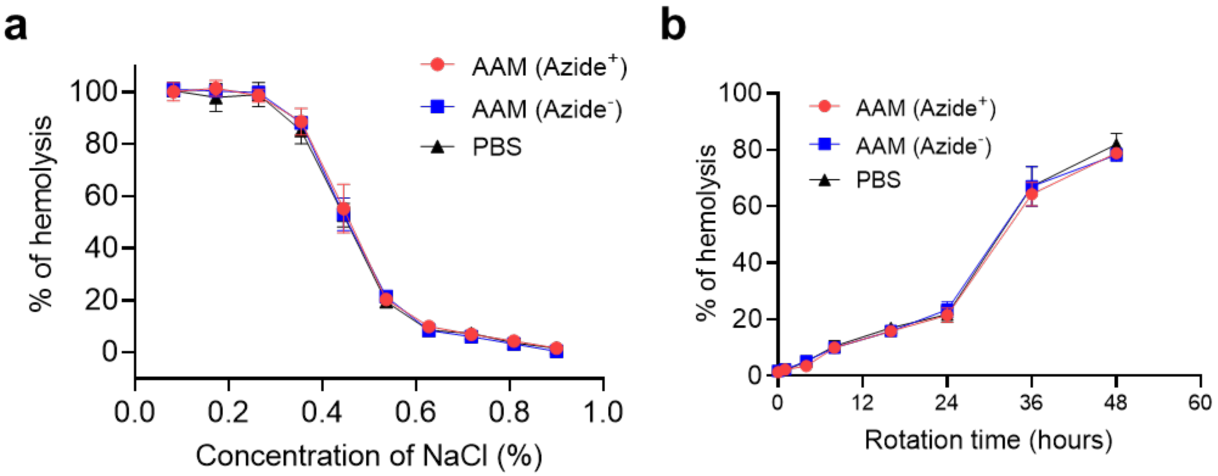

Supplementary Figure 6. In vivo metabolic glycan labeling of RBCs does not affect the osmotic and mechanical integrity of RBCs. C57BL/6 mice were i.v. injected with AAM or PBS twice daily for three consecutive days. Four days after the final injection, peripheral blood was collected and RBCs were isolated for functional assessment. (a) Osmotic fragility assay showing percentage of hemolysis as a function of NaCl concentration. (b) Mechanical fragility assay measuring hemolysis following rotational stress over time. Data are presented as mean ± SD. Hemolysis was calculated as the ratio of supernatant absorbance at 415 nm (hemoglobin) to the absorbance at 415 nm obtained from an equal number of RBCs fully lysed with the ACK lysis buffer. Source data for this figure is available in the Source Data file.

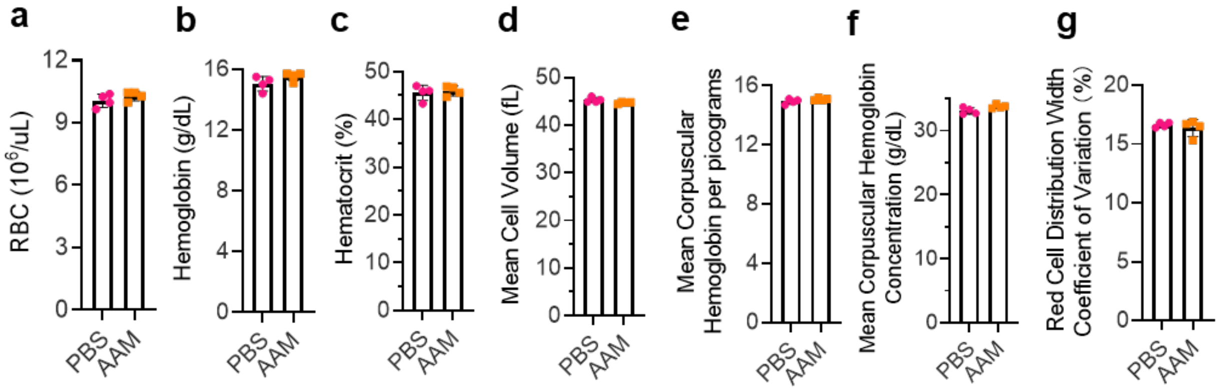

h

Morphology Feature	PBS-1	PBS-2	PBS-3	PBS-4	AAM-1	AAM-2	AAM-3	AAM-4
Polychromasia	1+	1+	1+	1+	1+	1+	1+	1+
Target Cells	Rare	–	–	–	–	–	–	–
Echinocytes	–	Rare	–	–	–	–	–	–
Hemoglobin Crystals	–	Rare	–	–	–	–	–	–
Howell–Jolly Bodies	–	–	–	–	Rare	Rare	Rare	Rare
Anisocytosis	–	–	–	–	Slight	Slight	Slight	–

Supplementary Figure 7. In vivo metabolic glycan labeling of RBCs does not alter hematological indices or morphology of RBCs. C57BL/6 mice were i.v. injected with AAM or PBS twice daily for three consecutive days. Four days after the final injection, peripheral blood was collected for hematological analysis. Quantified RBC parameters include (a) RBC count, (b) hemoglobin concentration, (c) hematocrit, (d) mean corpuscular volume (MCV), (e) mean corpuscular hemoglobin (MCH), (f) mean corpuscular hemoglobin concentration (MCHC), and (g) red cell distribution width (RDW). (h) Representative RBC morphology assessment for individual samples. All numerical data are presented as mean ± SD. For (a-g), n=4 mice per group. Source data for this figure is available in the Source Data file.

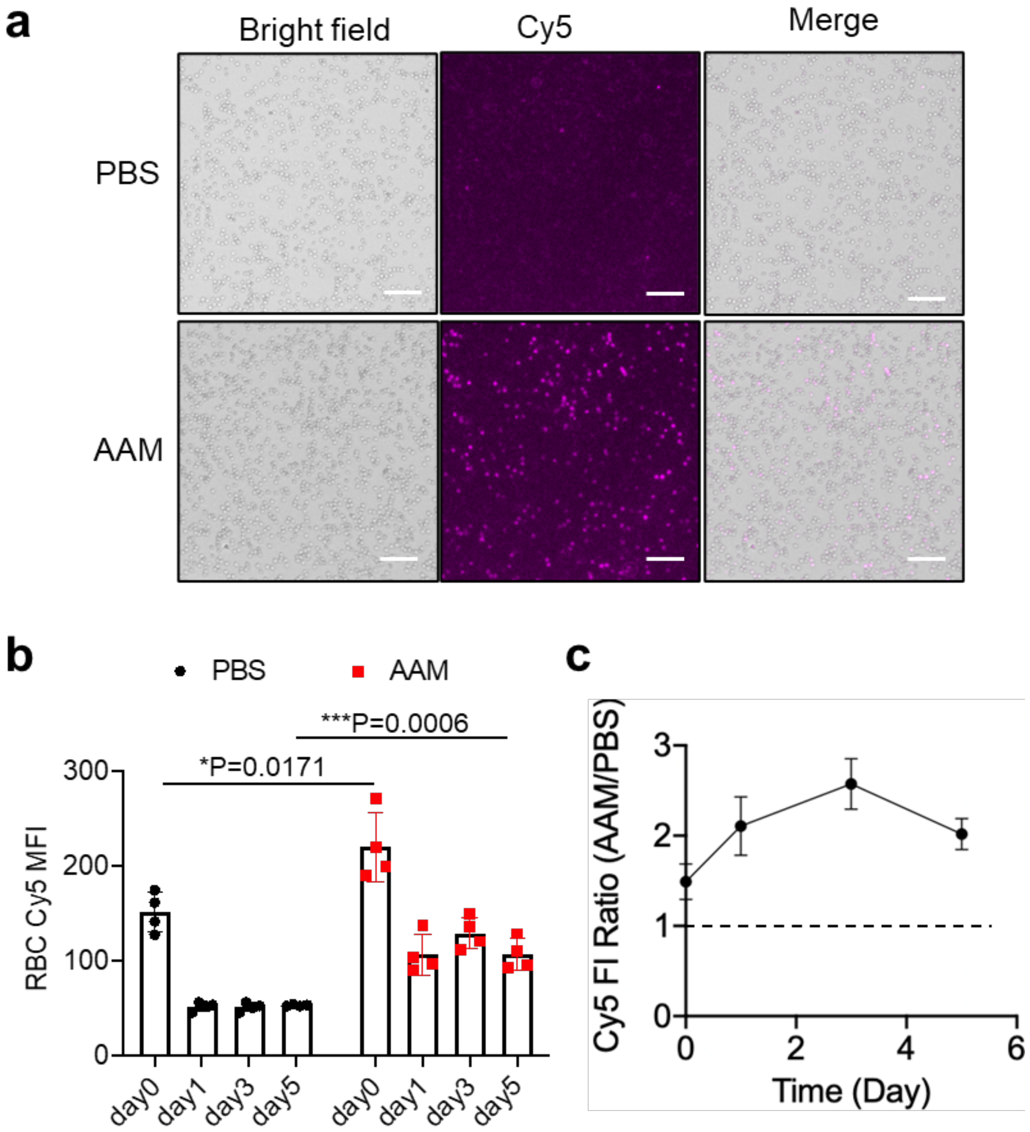

Supplementary Figure 8. DBCO-Cy5 conjugated to azido-labeled RBCs shows stable membrane retention. (a-c) AAM or PBS was i.v. injected into C57BL/6 mice twice daily for three days. After 7 days, RBCs were isolated and incubated with DBCO-Cy5 for 1 h ex vivo. After washing, RBCs were stored in the Alsever's solution for different times, prior to flow cytometry analysis. (a) Representative fluorescence images of RBCs isolated from AAM- or PBS-treated mice. Scale bar: 100 μm. (b) Cy5 fluorescence intensity of RBCs over time (n=4 mice per group). (c) Cy5 fluorescence intensity ratio of RBCs (AAM/PBS) in (b). All the numerical data are presented as mean ± SD (one-way ANOVA with post hoc Fisher's LSD test was used; 0.01 < *P ≤ 0.05; **P ≤ 0.01; ***P ≤ 0.001; ****P ≤ 0.0001). Experiment in (a) was performed at least three times independently with similar results. Source data for this figure is available in the Source Data file.

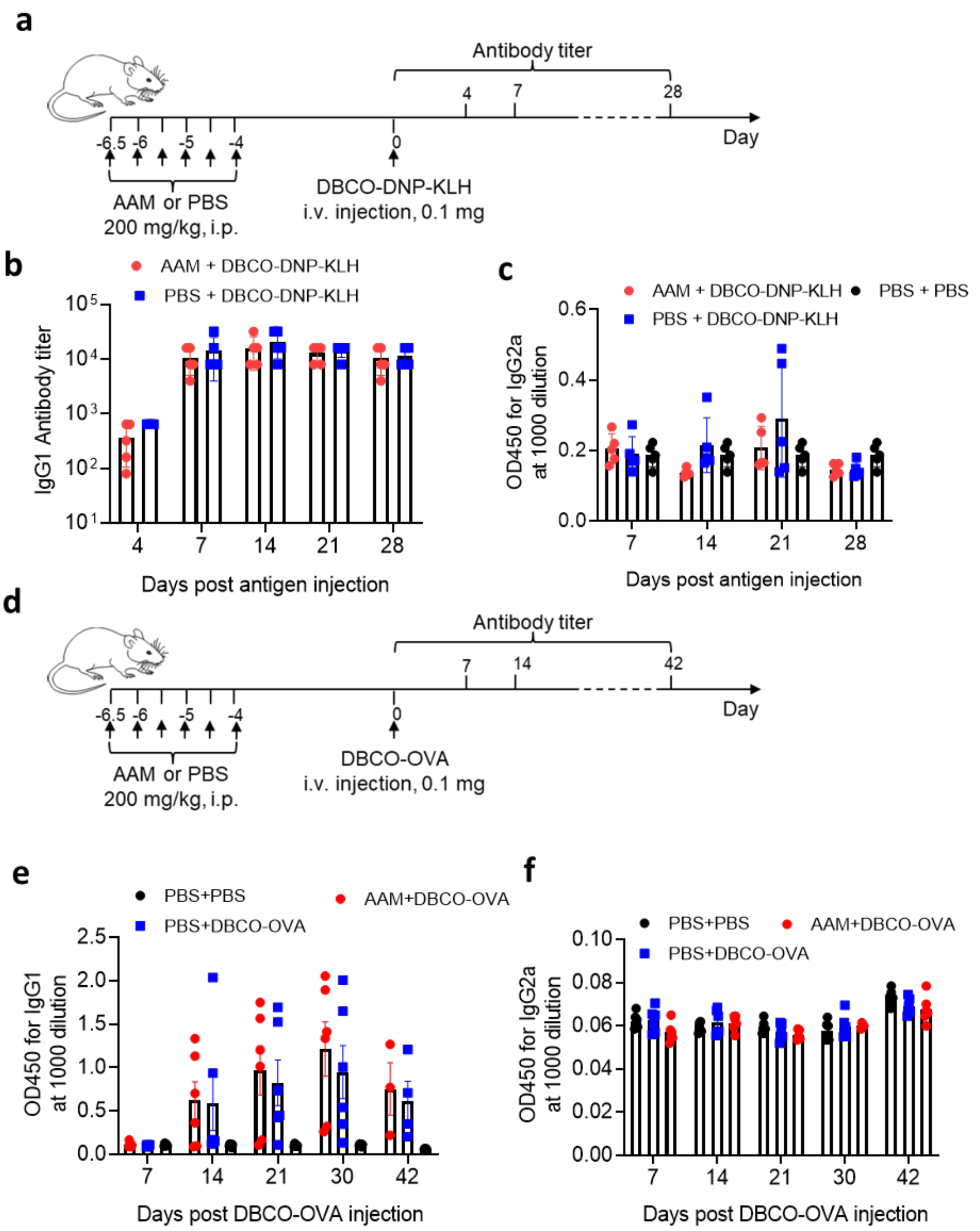

Supplementary Figure 9. In vivo targeting of antigens to circulating RBCs does not enhance the antibody responses. (a) Timeframe of DNP-KLH vaccination study. C57BL/6 mice were i.v. injected with AAM or PBS twice daily for three consecutive days. After labeling, mice were i.v. injected with DBCO-DNP-KLH, and serum antibody titers were monitored over time. Shown are anti-DNP-KLH (b) IgG1 and (c) IgG2a levels at indicated time points (n=4 mice per group). (d) Timeframe of ovalbumin (OVA) vaccination study. C57BL/6 mice were i.v. injected with AAM or PBS twice daily for three consecutive days. After labeling, mice were i.v. injected with DBCO-OVA, and serum antibody titers were monitored over time. Shown are anti-OVA (e) IgG1 and (f) IgG2a levels at indicated time points (n=6 mice per group). ELISA data are presented as OD_{450} values at the indicated serum dilution. All the numerical data are presented as mean ± SD. Source data for this figure is available in the Source Data file.

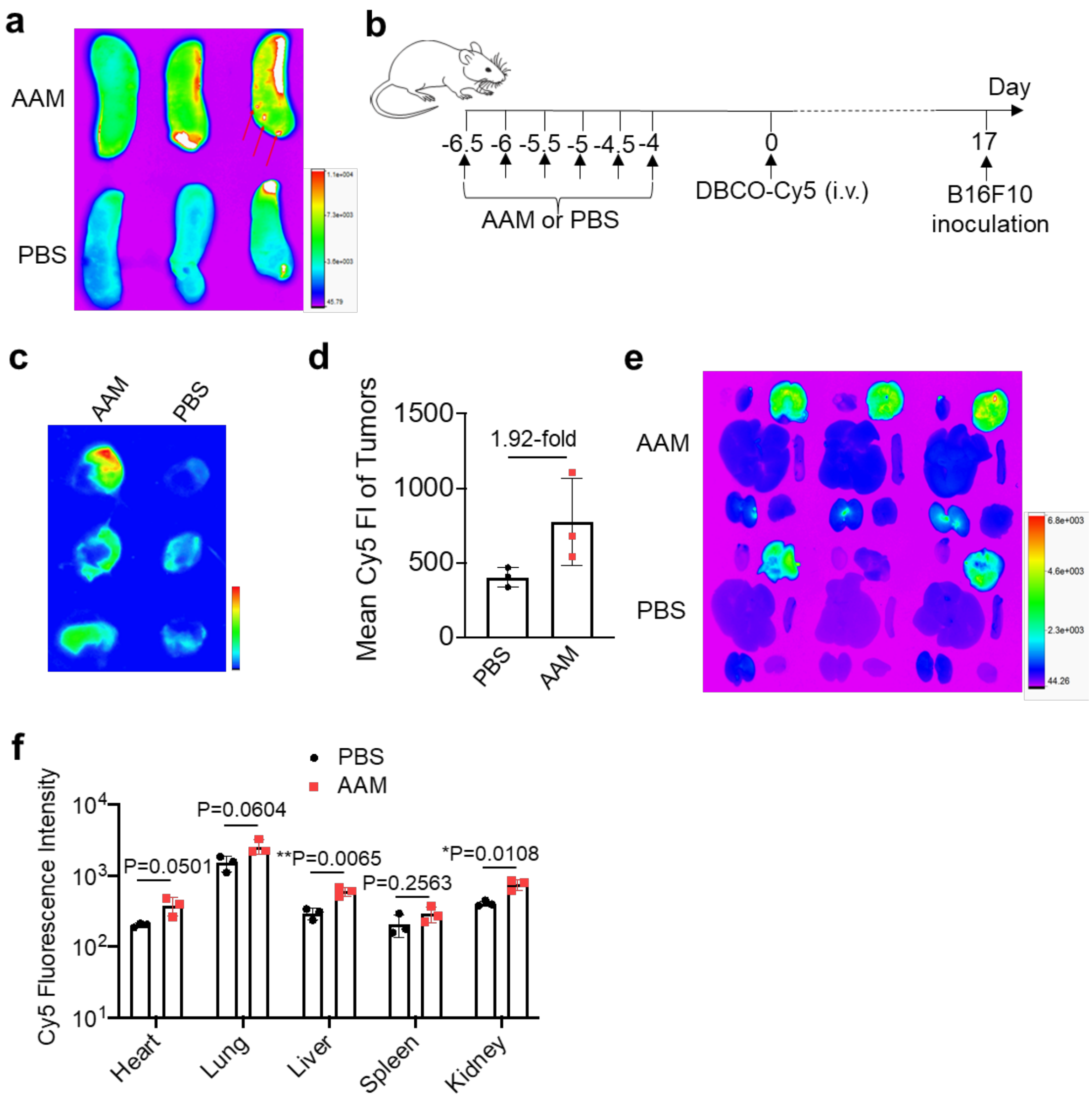

Supplementary Figure 10. In vivo conjugation of DBCO-Cy5 onto RBCs enables fluorescence imaging of blood vessels and tissues. (a) AAM or PBS was i.v. injected into Balb/c mice twice daily for three days (day -9.5 to day -7), followed by subcutaneous injection of 4T1 tumor cells on day -3 and i.p. injection of DBCO-Cy5 on day 0. Shown are IVIS images of spleens harvested from mice at 21 days post injection of DBCO-Cy5. The terminal arterioles structures in the spleen are indicated by the red arrows. (b-f) Timeframe for RBC labeling and B16F10 tumor imaging study. AAM or PBS was i.v. injected twice daily for three days, followed by i.v. injection of DBCO-Cy5 on day 0 and subcutaneous injection of B16F10 tumor cells on day 17. (c) IVIS imaging of B16F10 tumors at 7 days post tumor inoculation. (d) Quantified Cy5 fluorescence intensity of B16F10 tumors in (c) (n=3 mice per group). (e) IVIS imaging of major organs harvested from AAM- or PBS-treated mice on day 24. (f) Quantified Cy5 fluorescence intensity of organs from (e) (n=3 mice per group). All the numerical data are presented as mean ± SD (two-tailed Welch's t-test was used; $0.01 < {}^*P \leq 0.05$; $^{**}P \leq 0.01$; $^{***}P \leq 0.001$; $^{****}P \leq 0.0001$). Source data for this figure is available in the Source Data file.

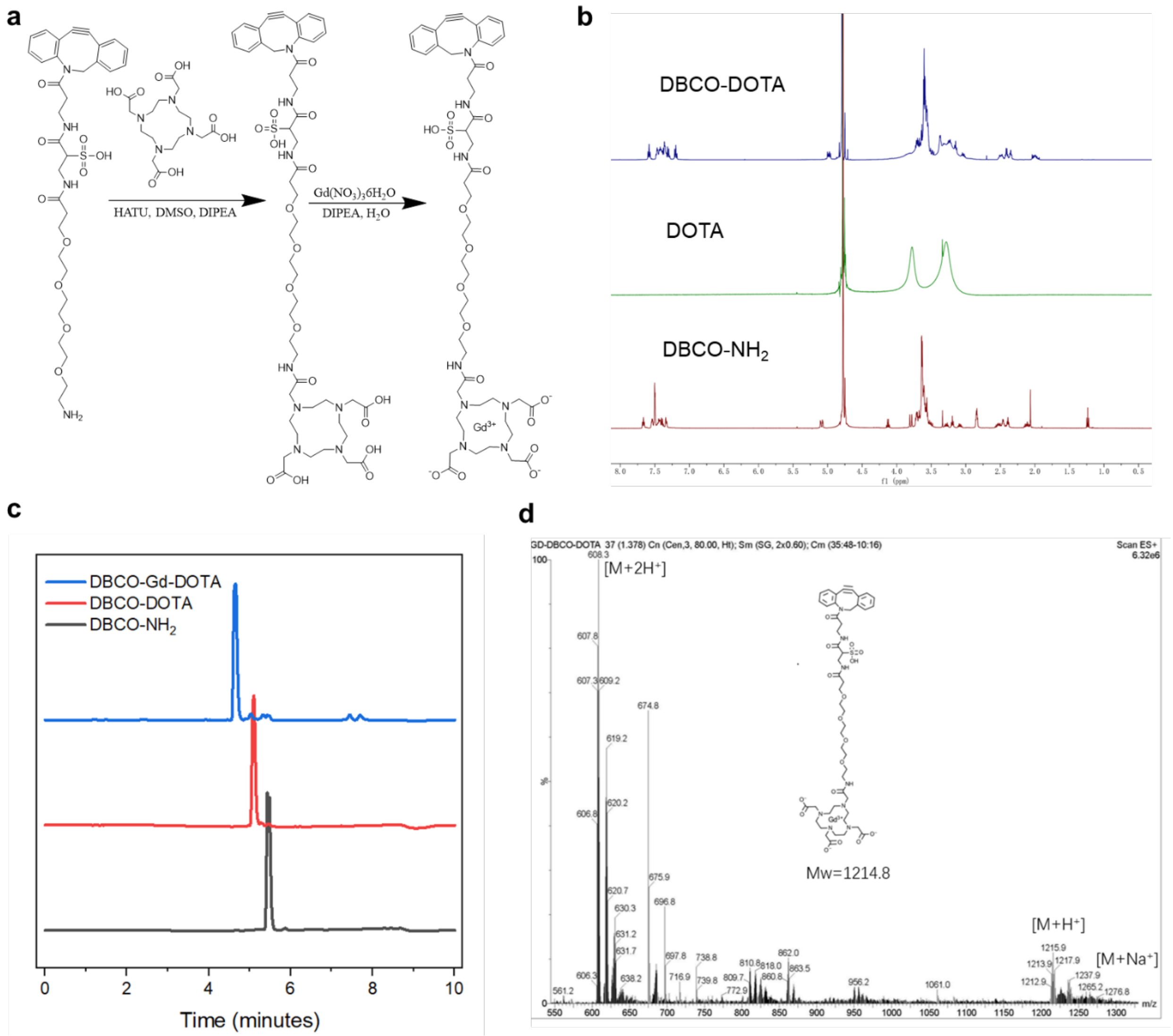

Supplementary Figure 11. Synthesis and characterization of DBCO-DOTA-Gd. (a) Synthesis route of DBCO-DOTA-Gd. (b) NMR spectra of DBCO-NH_2, DOTA, and DBCO-DOTA in D_2O. (c) HPLC profiles of DBCO-NH_2 (black), DBCO-DOTA (red), and DBCO-DOTA-Gd (blue). The detection wavelength was set at 310 nm. (d) Mass spectrum of DBCO-DOTA-Gd. The peaks for at $[M+H]^+$, $[M+2H]^{2+}$ and $[M+Na]^+$ can be found. Source data for this figure is available in the Source Data file.

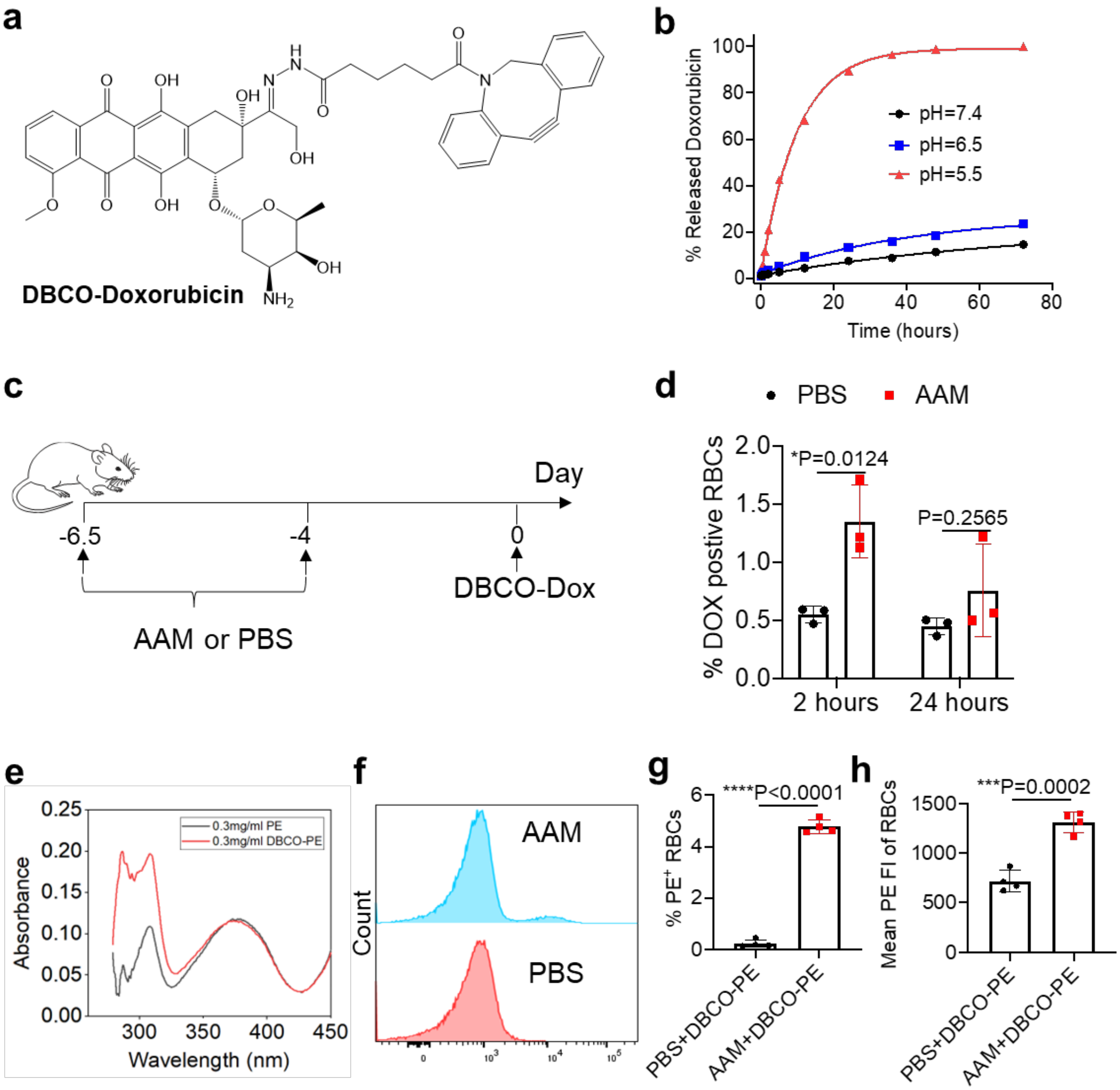

Supplementary Figure 12. Azido-tagged RBCs enable conjugation of small-molecule and macromolecular cargos in vitro and in vivo. (a) Synthesis of acid-labile DBCO-doxorubicin (DBCO-Dox). (b) Release kinetics of DBCO-Dox at different pH, as measured by HPLC. (c-d) C57BL/6 mice were i.v. injected with AAM twice daily for three days, and DBCO-Dox was i.v. injected at 4 days post the last AAM injection. The blood was collected after 2 or 24 h post DBCO-Dox injection. (c) Timeframe of study. (d) Percentages of Dox-containing RBCs at 2 or 24 h post DBCO-Dox injection (n=3 mice per group). (e) UV spectra of PE and DBCO-functionalized PE. (f-h) Flow cytometry analysis of in vitro conjugation of PE to RBCs. RBCs were collected from AAM-treated mice at 14 days post AAM injection and incubated with DBCO-PE for 2 h. (f) Representative PE histogram of RBCs. (g) Percentages of PE-containing RBCs. (h) Mean PE fluorescence intensity of RBCs. All the numerical data are presented as mean ± SD (two-tailed Welch's t-test was used; $0.01 < {*P} \leq 0.05$; $**P \leq 0.01$; $***P \leq 0.001$; $****P \leq 0.0001$). For (g-h), n=4 mice per group. Source data for this figure is available in the Source Data file.

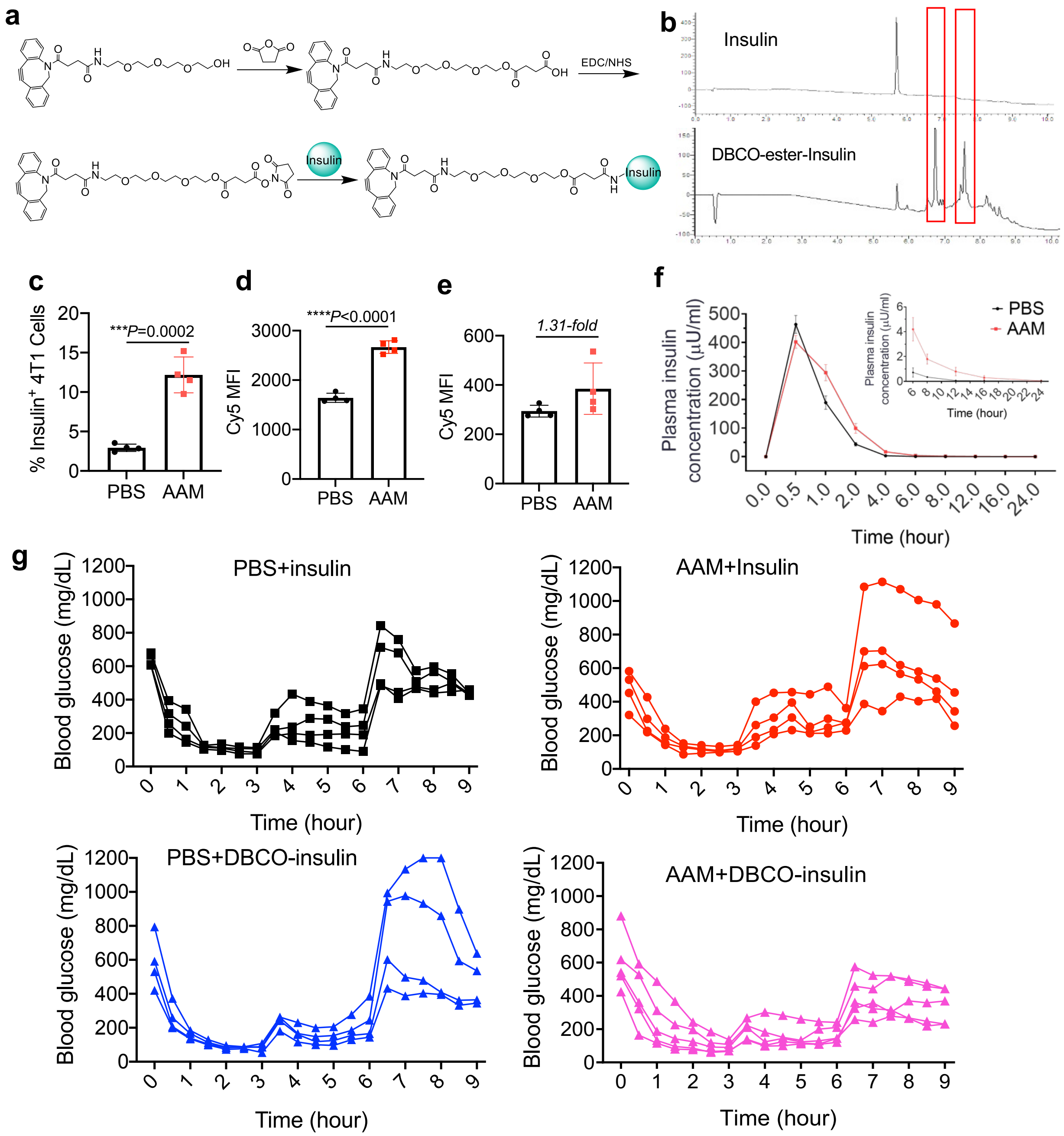

Supplementary Figure 13. DBCO-insulin can conjugate to azido-labeled RBCs in vivo for improved blood glucose control. (a) Synthesis route of DBCO-insulin with an ester linkage. (b) HPLC profiles of insulin and DBCO-insulin with a detection wavelength of 280 nm. Peaks for DBCO-insulin are indicated in red rectangular. (c-d) 4T1 cells (n=4 samples) were treated with AAM or PBS for 24 h, followed by 1-h incubation with DBCO-insulin. Cell-surface insulin was detected by staining with rabbit anti-insulin and Cy5-conjugated goat anti-rabbit secondary antibody. Shown are (c) percentages of insulin-positive 4T1 cells and (d) Mean Cy5 fluorescence intensity of 4T1 cells. (e) Mean Cy5 fluorescence intensity of RBCs (n=4 mice per group). C57BL/6 mice were i.v. injected with AAM or PBS twice a day for three days. After 14 days, RBCs were isolated and incubated with DBCO-insulin for 1 h. Cell-surface insulin was detected

by staining with rabbit anti-insulin and Cy5-conjugated goat anti-Rabbit secondary antibody. (f) Blood concentration of insulin at different times post i.p. injection of DBCO-insulin (10 IU/kg) in AAM-treated or PBS-treated mice. (g) Blood glucose levels of individual mice for each group during the course of the glucose tolerance test. Mice were fasted for 12 h, and then 10 IU/kg DBCO-insulin or insulin was i.p. injected. Glucose was i.p. injected at 3 and 6 h. All the numerical data are presented as mean ± SD (two-tailed Welch's t-test was used; $0.01 < {}^{*}P \leq 0.05$; $^{**}P \leq 0.01$; $^{***}P \leq 0.001$; $^{****}P \leq 0.0001$). Source data for this figure is available in the Source Data file.
